# Relationships between physical fitness characteristics, technical skill attributes, and sports injury in female Australian football players

**DOI:** 10.1371/journal.pone.0298267

**Published:** 2024-02-22

**Authors:** Jessica B. Farley, Miranda O’Hara, Justin W. L. Keogh, Carl T. Woods, Evelyne Rathbone, Nikki Milne

**Affiliations:** 1 Faculty of Health Sciences and Medicine, Bond Institute of Health and Sport, Bond University, Gold Coast, QLD, Australia; 2 Brisbane Lions Women’s Australian Football Club, Brisbane, QLD, Australia; 3 Restore Function Physiotherapy, Greenslopes, QLD, Australia; 4 Sports Performance Research Centre New Zealand, AUT University, Auckland, New Zealand; 5 Kasturba Medical College, Mangalore, Manipal Academy of Higher Education, Manipal, Karnataka, India; 6 Institute for Health and Sport, Victoria University, Melbourne, VIC, Australia; Università degli Studi di Milano: Universita degli Studi di Milano, ITALY

## Abstract

**Objectives:**

To explore the relationships between physical fitness and i) technical skills and ii) time-loss from Australian football injury in female players across the talent and participation pathways.

**Methods:**

This study uses a subset of data from two cross-sectional and one prospective cohort studies. A total of 223 female Australian football players across five competition levels (elite/non-elite senior, high-level junior, and non-elite junior (14–17 years)/(10–13 years)) were included in this study. Comprehensive physical fitness assessments and modified Australian football kicking and handballing tests were conducted in the 2018/19 pre-seasons. During the respective competitive in-season, time-loss injuries were recorded by team personnel. Stepwise multiple linear regressions were performed to determine the relationship between physical fitness and kicking and handballing scores. Cox proportional regressions were conducted to identify physical fitness factors associated with injury.

**Results:**

Increased running vertical jump height, greater hip abduction strength, and faster timed 6 m hop speed demonstrated a relationship with kicking accuracy when adjusted for years of Australian football playing experience (adjusted *R*^*2*^ = 0.522, *p* < 0.001). Faster agility time and increased lean mass were associated with better handballing accuracy (adjusted *R*^*2*^ = 0.221, *p* < 0.001). Multivariate Cox regression revealed an increased risk for sustaining a time-loss injury in less agile players (adjusted HR 2.41, 95% CI 1.23, 4.73, *p* = 0.010). However, this relationship no longer remained when adjusted for age and years of Australian football experience (adjusted HR 1.68, 95% CI 0.81, 3.50, *p* = 0.166).

**Conclusions:**

Physical fitness may be a significant factor contributing to development of Australian football technical skills in female players. However, its role is unclear in protecting against injury risk in this athlete population. Further research is needed to explore the multifactorial and complex phenomenon of talent development and injury risk reduction in female Australian football players.

## Introduction

Australian football (AF) is a dynamic, team invasion sport, requiring players to possess a blend of capacities and skills [1]. For example, competing in AF requires players to perform various athletic movements, such as high-speed running and jumping, and technical skills, like kicking and handballing, to maintain possession and move the football down-field in an attempt to score a goal [1]. At an individual level, players train regularly to develop their athletic capacities and skills with the aim to remain free from injury. Additionally, from a team tactical perspective, sports injury can negatively impact team sporting success (e.g., by reducing player availability) [2]. As such, minimising injury risk is of importance to ensure player health and well-being, as well as improve sporting outcomes. The inauguration of the Australian Football League Women’s (AFLW) competition in 2017 has resulted in a rapid rise in female participation in the sport [3]. Due to this expansion, there is an urgency for the research community to respond to support female AF and accompanying sport practitioners, coaches, and athletes, as they may be utilising evidence to inform talent development and injury risk reduction programs from the men’s AF literature. Given the known sex differences demonstrated in team ball sports in technical skill performance [4–6], sports injury risk [7–9], and female sporting environments [10, 11], this may not be appropriate.

Physical preparation is an integral component in the success or failure of sporting outcomes [12]. Physical fitness can be defined as a set of characteristics that an individual has or develops relating to their ability to perform physical activity [13]. Physical fitness encompasses a range of components, including body composition, cardiorespiratory fitness, muscular strength, muscular endurance, flexibility, agility, balance, coordination, power, reaction time, and speed [13]. These measurable characteristics can be considered inter-related [14] and have been shown to have a significant positive association with improved outcomes in physical activity, such as sport participation [15]. In men’s AF, unique physical fitness combinations of speed, repeated sprint ability, change of direction, power, cardiorespiratory fitness, muscular strength, and movement quality are required across participation and talent pathways [16]. Initial research in senior female AF competition levels have examined physical game demands, indicating cardiorespiratory fitness and speed are important physical fitness characteristics that allow athletes to meet match demands and enable enhanced performance [17–22]. Kicking and handballing skills are also integral components to the game, with one study reporting no positional differences in the number of kicks and handballs performed during elite senior female competition [17], suggesting that they are essential skills for all players irrespective of position. Additionally, the physical characteristics [23] and kicking and handballing skills [24] of female AF players have been profiled across five competition levels, which included junior and senior cohorts participating in talent and community competitions. Whilst the above-cited research has provided preliminary insight to better understand the physical and technical attributes characteristic of match-play or a snapshot of players, little is known about the relationships between physical fitness and technical skills in female AF players.

Understanding the associations between physical fitness and technical skills may be important when designing talent development programs to enhance sport performance outcomes in women’s and girls’ AF competitions. Joseph and colleagues [25], for example, noted that junior male AF players with greater cardiorespiratory fitness may be better equipped to maintain kicking speeds over the course of a game, which may enhance scoring opportunities. Hart and colleagues [26] showed accurate sub-elite male AF kickers had greater relative lean mass, muscular strength, and bilateral symmetry between the lower extremities compared to inaccurate kickers. In a recent systematic review investigating the relationships between physical fitness and technical skills in female, team ball sport athletes (including handball, volleyball, soccer, basketball, netball, lacrosse, and softball), three clear associations were found between i) body composition and defensive technical skills, ii) agility and movement with a ball, and iii) coordination and movement with a ball, highlighting these physical fitness components may be of importance when developing technical skill in female team ball sport athletes [27]. However, likely due to the infancy of the AFLW competition, no AF studies were included in this systematic review, indicating the relationships between physical fitness and technical skills in this athlete population is currently unknown.

In addition to mediating performance development strategies, profiling specific sporting populations by linking competition demands with skill competency and physical fitness can potentially provide insight into injury risk factors [28]. For example, in elite junior male AF players, faster acceleration indicated by 5-m sprint times have been shown to be associated with injury (Risk Ratio = 0.013, 95% CI 0.00, 0.44, p = 0.016), suggesting that faster players may be involved in more game actions and as such, exposed to more inciting injury events [28]. Additionally, coaches strategically coordinate their available players fit to perform in order to maximise their team’s performance [29]. Therefore, ensuring that injury risk factors are managed appropriately through injury risk reduction programs to minimise the number of injured athletes is also of great importance to sporting performance [29]. Research has indicated that time-loss injuries, defined as an event which resulted in the player missing their usual training session or match participation [30], are common in female AF players [31]. Many injuries recorded in a prospective study were deemed to be moderate severity (8–28 days missed) [31], which has implications on player availability. For example, 8–28 days missed equates to one to four games missed, or up to almost half of a regular ten game in-season in the elite senior female competition (AFLW). Given the consequences of injuries impacting an athlete’s wellbeing and team selection availability, research to support injury risk reduction programs is warranted to support the rapidly expanding participation of female AF.

One such response to the urgent need to develop contextually relevant injury risk reduction programs for female AF populations is the creation of Prep-to-Play PRO (professional), which is an anterior cruciate ligament (ACL) injury risk reduction program for women playing in the AFLW [32]. This program has also been adapted for women’s and girls’ community AF (Prep-to-Play) [33]. Prep-to-Play PRO was developed in partnership with the sport’s governing organisation and engaged sport practitioners and AFLW players [32], which revealed five key elements for inclusion: football-specific preparation, movement skills, strength and conditioning, individual preparation, and education [34]. While physical fitness is captured within these elements, empirical evidence of its validity is still to be achieved [35]. Namely, this intervention development process [32] has been aligned with step 3 (develop preventive measure) of the Translating Research into Injury Prevention Practice Framework [36]. While this is an exemplar of co-design with end users and content and context experts across the socio-ecological levels within female elite AF [32], the second step in injury risk reduction research is to understand risk factors [36] or risk profile regularities [37]. Identifying risk factors can be the first step to indicating causal relationships [38].

One study has explored ACL injury situations in elite female AF players [39]. Video analysis of 21 ACL injuries sustained during match-play during the 2017–2020 AFLW seasons revealed the most common mechanisms of injury included performing a sidestep cutting manoeuvre, typically when applying defensive pressure [39]. Rearfoot footfall, knee valgus collapse, and extended knee postures were also common AFLW ACL injury characteristics [39]. These findings may provide preliminary insight into how ACL injuries occur in this athlete population; however, many risk factors are currently speculative and further research is warranted to suggest potential causal links [35]. Additionally, this initial aetiology research has been targeted to only ACL injuries at the elite female competition level. Whilst a top injury priority, understanding associations between physical fitness characteristics and overall injury risk across the talent and participation pathways may provide evidence to support or enhance Prep-to-Play and Prep-to-Play PRO as a general injury risk reduction program.

Physical fitness is one element of talent development and injury risk reduction programs. A variety of physical fitness characteristics can support players in meeting game demands and enhance transition between competition levels [40], and it is a key component to athlete development, which may also have benefits for reducing injury risk [41, 42]. Given the lack of research evidence regarding injury risk factors and aspects to influence technical skill development in female AF players, investigation to fill these gaps is warranted. Understanding potential causal links can guide future research to inform the design of targeted injury risk reduction and talent development programs; with the end goal to support longevity of female AF players whilst maximising sporting outcomes. Therefore, the aims of this study were to explore the relationships between physical fitness and i) technical skill attributes and ii) time-loss injury in female AF players.

## Materials and methods

### Study design and participants

An observational prospective cohort design was conducted over competitive seasons in either 2018 and 2019 to explore the relationships between physical fitness characteristics, technical skill attributes, and sports injury. Participant recruitment began on November 2, 2017, and data collection ceased on September 8, 2019. This study uses a subset of data from two cross-sectional studies that profiled physical fitness characteristics [23] and kicking and handballing technical skills [24], as well as one prospective cohort study that examined injury profiles [31] in female AF players across five competition levels. A subset of data from 240 female AF players from southeast Queensland from these three studies were utilised for this study. To be included in this study, players were participating in regular training sessions for at least two weeks with an AF team registered in a female competition and had to have completed the physical fitness and technical skills testing and/or the player’s team provided injury data in the same data collection year. Seventeen players did not meet this eligibility criteria, therefore, a total of 223 female AF players were classified into five, previously defined competition levels [23]: elite senior (≥18 years participating in the AFLW competition or selected into a state representative team or talent academy program) (*n* = 53), non-elite senior (≥18 years participating in one of the state community competitions) (*n* = 57), high-level junior (<18 years and selected into a state representative team or talent academy program) (*n* = 34), non-elite junior (14–17 years and playing for a school or community club team) (*n* = 37), and non-elite junior (10–13 years and playing for a school or community club team) (*n* = 42). All players received an explanatory statement of the study, volunteered to participate, and provided written informed consent. The Bond University Human Research Ethics Committee approved the study (16116).

### Procedures

The procedures for collecting physical fitness characteristics, technical skill attributes, and sports injury data for this study have been published elsewhere [23, 24, 31]. Players underwent a testing battery during the pre-season for their subsequent playing season. The testing battery consisted of 28 different physical fitness assessments [23] and two technical skills tests [24] following standardised testing protocols in the literature. Over the course of each competitive season, injuries were recorded prospectively by designated team personnel, such as the team physiotherapist or sport trainer, using methods previously described [31]. Injury data and AF exposure were provided to the primary author by appropriate team personnel (e.g., team medical and coaching staff) at the end of season, combined with publicly accessible data from SportsTG website (websites.sportstg.com; now rebranded as PlayHQ) [31]. A summary of the data collection procedures is provided in Table 1.

**Table 1:** Summary of data collection procedures to obtain relevant physical fitness, technical skill, and sports injury data utilised in the analysis.

	Physical Fitness	Technical Skill	Sports Injury
Procedures previously published	Farley et al. [23]	Farley et al. [24]	Farley et al. [31]
Year study conducted	Pre-season of the 2018 and 2019 competitive seasons	Pre-season of the 2018 and 2019 competitive seasons	Pre-season and in-season of the 2018 and 2019 competitive seasons
Length of study	Across 2–3 sessions; cohorts did not complete testing at the same time	Across 1–2 sessions within one week; cohorts did not complete testing at the same time	Pre-season through the end of the same competitive season; as the seasons differed across the five levels, each cohort did not complete data collection at the same time of the year
Most common setting	Grass field and gym	Grass field and gym	Team training and match day environments
Pre-testing procedures	No dietary or exercise restrictions were implemented; players completed a self-directed warm-up for 10–15 minutes prior to testing	No dietary or exercise restrictions were implemented; players completed a self-directed warm-up for 10–15 minutes prior to testing	N/A
Data assessors	All assessors attended a familiarisation training session prior to data collection	All assessors attended a familiarisation training session prior to data collection	Club personnel received written instructions for recording injuries
Data collection	28 physical fitness assessments encompassing: body composition, flexibility, muscular strength, muscular endurance, balance, reaction time, cardiovascular fitness, agility, coordination, power, and speed	Modified Australian football kicking and handballing tests	Medical attention* and time-loss injuries recorded over one competitive season

*Injuries requiring medical attention may also result in time lost from training and/or match participation

Based on the results of the studies outlined in Table 1, a selection of explanatory and outcome variables was included in the analysis for the present study (Table 2). A subset of 36 physical fitness variables were chosen that represented the comprehensive physical fitness components profile as previously identified [23]. Due to the potential floor effect in the modified Australian football handballing test (mAFHT) for the non-dominant side previously reported [24], only dominant modified Australian football kicking test (mAFKT) and dominant mAFHT scores were selected for analysis to represent these sport-specific technical skills. Lastly, Farley and colleagues [31] reported injuries resulting in time missed were most frequent in female AF players. As in-season injuries can directly influence player selection and team performance [2], only injuries that were sustained in-season due to any mechanism of injury while participating in an AF training or match and resulted in the player missing their usual AF participation were analysed. Age and years of AF experience were extracted from a dataset using a pre-data collection questionnaire used in the physical fitness profiling study [23].

**Table 2:** Physical fitness, technical skill, and sports injury variables included in the analysis.

Explanatory variables	
Body composition	Body mass (kg), bone mineral density (g/cm^2^), hand span (cm), height (cm), lean mass (kg), body fat percentage (%)
Flexibility	Sit-and-reach distance (cm), dominant knee extension angle (degrees); dominant and non-dominant weight-bearing lunge test (cm), weight-bearing lunge test limb difference (cm)
Muscular strength and endurance	Dominant hand grip strength (kg), dominant shoulder internal rotation strength (N), dominant hip abduction strength (N), dominant knee extension strength (N), dominant knee flexion strength (N), adductor squeeze test (hips positioned at 45 degrees flexion) (mmHg), dominant and non-dominant hamstrings/quadriceps ratios, dominant side bridge (s), dominant/non-dominant side bridge ratio, dominant and non-dominant single leg calf raises (repetitions)
Balance	Dominant anterior reach (cm), non-dominant anterior reach (cm), dominant posteromedial reach (cm), dominant posterolateral reach (cm), dominant composite reach score (%)
Reaction time	Audio inverse efficiency score (ms), visual inverse efficiency score (ms)
Whole-body locomotor performance	Yo-Yo intermittent recovery test (level 1) distance (m), AFL agility test (s), 20-m sprint (s), non-dominant running vertical jump (cm), dominant 6 m hop (s), 6 m hop limb symmetry index ratio
**Outcome variables**	
Technical skill	Dominant modified Australian football kicking test score, dominant modified Australian football handballing test score
Sports injury	An injury event that occurred during the competitive season resulting in time lost from Australian football participation

AFL = Australian Football League

### Statistical analysis

Descriptive statistics were reported as mean ± standard deviation (SD) for normally distributed continuous data and median (IQR) for skewed data. Normality was established by the Shapiro-Wilk test and inspection of histograms and normal Q-Q plots. Categorical data were reported as frequencies and percentages. Due to the small sample size in this study, a principal component analysis (PCA) was utilised to reduce dimensionality and establish optimal sets of key physical fitness variables to use in regression analyses. Eigenvalues of ≥1.0 and visual inspection of scree plots were used to determine a suitable number of principal components [43]. To explore the relationship between physical fitness and the two technical skill tests (mAFKT and mAFHT), two stepwise multiple linear regression models were built on the entire sample and for each competition level. From the original 223 female AF players recruited in this study, a total of 184 players (elite senior: n = 19; non-elite senior: n = 57; high-level junior: n = 29; non-elite junior (14–17 years): n = 37; non-elite junior (10–13 years): n = 42) were eligible to be included in the stepwise multiple linear regression analyses, as some players (*n* = 39) did not complete the technical skill testing due to data collection constraints, such as weather, or team-limiting constraints, such as load management. Unstandardised and standardised coefficients, 95% CIs, standard errors of the estimate, and *p*-values were reported along with the *R*^*2*^ and adjusted *R*^*2*^ for each model.

To examine the relationship between physical fitness and in-season, time-loss AF injury, univariate Cox proportional hazards regression models were built. Four teams did not provide injury data at the end of their respective season, resulting in a total of 148 players (elite senior: n = 39; non-elite senior: n = 22; high-level junior: n = 28; non-elite junior (14–17 years): n = 25; non-elite junior (10–13 years): n = 34) across the five competition levels eligible for the sports injury analysis. Variables deemed significant from the univariate analyses were then entered into a multivariate Cox proportional hazards regression model [44]. Time to injury was defined as completed exposure hours, indicated by the number of hours participated in AF training and matches between the start of Round 1 of the competitive season and the date of injury or censored date. Players were considered censored if they were not injured by completion of the competitive season or if they quit the team. Each potential risk factor variable was grouped into identified categories relevant in the literature or dichotomised according to the median value to reduce the likelihood of small counts in the model [45]. Standard Kaplan-Meier survival curves were also conducted to explore the probability of remaining injury free during the competitive season across competition levels. Age and years of AF experience were considered as potential confounding variables and adjusted for in the regression analyses. Data were analysed using SPSS (Version 26) and statistical software R (Version 3.6.3) [46]. The level of statistical significance was set at *p* < 0.05 for all analyses.

## Results

Five principal components were retained by the PCA, explaining 58.8% of the variance of the original physical fitness dataset. The first component accounted for 29.3% of the variance and represented muscular strength and performance-related physical fitness (including, power, coordination, agility, and speed). The second and third components accounted for 10.2% and 8.7% of the variance and predominantly represented balance, namely centre of gravity and ankle mobility elements, respectively. The fourth component described 5.8% of the variance and characterised paired limb measures assessing aspects of between limb symmetry, while the fifth component (4.8% of the variance) predominantly concerned lower extremity flexibility. As Component 4’s pattern represented measures conducted on both limbs, rather than characterising physical fitness, the contribution of these variables was not included in subsequent analyses as this was not of interest in this study. A total of 13 most influential physical fitness variables were identified (Table 3).

**Table 3:** The five retained principal components and their correlations with the 13 most influential potential physical fitness explanatory variables.

Potential explanatory variables	Principal component				
	1	2	3	4	5
**Variance explained**	**29.3%**	**10.2%**	**8.7%**	**5.8%**	**4.8%**
20 m sprint time	-0.74	0.29	0.19	0.13	-0.18
6 m hop time	-0.67	0.37	0.23	-0.00	-0.03
AFL agility time	-0.72	0.33	0.23	0.10	-0.01
Body fat percentage	-0.45	0.66	0.21	0.08	-0.19
Hip abduction strength	0.78	0.24	-0.22	0.11	-0.01
Knee extension strength	0.66	0.36	0.18	-0.22	-0.08
Knee extension angle	-0.27	-0.03	-0.24	-0.10	0.68
Knee flexion strength	0.66	0.09	-0.20	0.30	-0.06
Lean mass	0.85	0.38	-0.02	-0.02	0.02
Running vertical jump	0.73	-0.23	-0.20	-0.15	0.11
Shoulder internal rotation strength	0.75	0.11	-0.25	0.01	-0.07
Y-balance anterior reach distance	0.36	-0.18	0.68	0.01	0.22
Y-balance composite reach score	0.14	-0.67	0.47	0.04	-0.30

All muscular strength measures, knee extension angle, Y-balance composite reach score, and 6 m hop time represent dominant side only. Y-balance anterior reach and running vertical jump tests indicative of non-dominant limb. AFL = Australian Football League.

Dominant side bridge and visual inverse efficiency score variables were not represented in the most influential variables from the main principal components but were still of interest, as they measure muscular endurance and reaction time components of physical fitness, respectively. As such, they were considered in subsequent analyses to provide a comprehensive physical fitness profile. The final set of physical fitness characteristics, as well as demographic data, technical skill attributes, and injury frequency of the included players within each competition level utilised in the regression analyses are presented in Table 4.

**Table 4:** Demographic data, physical fitness characteristics, and technical skill and sports injury outcomes of included female Australian football players.

Variables	n	Elite senior (≥18 years)	n	Non-elite senior (≥18 years)	n	High-level junior (<18 years)	n	Non-elite junior (14–17 years)	n	Non-elite junior (10–13 years)
Age (years)	53	22.1 (6.7)	57	22.9 (6.2)	34	17.2 (1.2)	37	15.9 ± 0.9	42	12.5 ± 1.0
Playing experience (years)	50	6.6 ± 4.5	57	2.0 (3.0)	34	3.5 (3.0)	33	1.8 ± 1.1	37	1.0 (1.0)
**Body composition**										
Body fat (%)	49	27.9 ± 5.0	49	29.8 ± 6.4	26	28.3 ± 3.7	30	32.7 ± 5.3	40	31.0 ± 6.5
Lean mass (kg)	49	46.3 ± 6.1	49	44.6 ± 6.2	26	41.6 ± 3.8	30	37.8 ± 4.6	40	31.6 ± 6.6
**Muscular strength and endurance**										
Shoulder IR strength (N)	52	157.1 ± 36.8	43	129.9 ± 34.8	32	136.3 ± 31.0	26	101.3 ± 21.1	40	84.5 (24.7)
Hip ABD strength (N)	49	145.8 ± 33.6	44	112.4 ± 27.4	33	136.3 ± 40.9	27	92.2 ± 17.9	40	75.2 ± 24.4
Knee EXT strength (N)	45	324.7 ± 62.1	44	298.1 ± 102.8	33	302.3 ± 72.3	27	306.3 ± 85.9	40	211.8 ± 60.9
Knee FLEX strength (N)	49	151.6 ± 35.6	44	115.6 (38.8)	33	130.1 ± 36.8	27	127.6 ± 28.5	40	100.0 (32.8)
Side bridge (s)	48	102.0 (60.1)	50	77.6 ± 32.8	32	70.5 (23.5)	37	58.8 ± 21.6	41	48.3 ± 27.2
**Flexibility**										
Knee EXT angle (deg)	50	10.0 ± 9.0	52	13.0 ± 12.0	32	14.0 ± 11.0	37	0.0 (8.0)	42	16.0 ± 14.0
**Balance**										
Y-balance composite reach (%)	51	102.5 ± 6.4	51	101.3 ± 7.5	32	98.1 ± 7.2	31	103.6 ± 5.9	41	100.6 ± 10.1
Y-balance ANT reach (cm)	52	60.9 ± 7.6	53	60.9 ± 7.4	32	60.1 ± 5.8	30	62.2 ± 5.7	41	60.5 ± 5.8
**Reaction time**										
VIES (ms)	48	370.0 ± 58.0	52	398.0 ± 48.0	32	381.0 ± 41.0	35	397.0 (79.0)	41	434.0 (135.0)
**Whole-body locomotor performance**										
Running vertical jump (cm)	39	52.2 ± 7.7	49	46.0 ± 10.6	23	52.4 ± 8.4	37	39.4 ± 8.5	40	33.9 ± 10.9
20 m sprint (s)	41	3.37 ± 0.15	47	3.48 (0.25)	20	3.40 ± 0.16	27	3.58 ± 0.23	39	3.78 ± 0.39
AFL agility (s)	26	8.96 ± 0.35	52	8.98 (0.65)	25	8.82 ± 0.29	33	9.88 ± 0.66	40	10.23 ± 0.85
6 m hop (s)	24	1.95 ± 0.18	49	2.07 (0.31)	27	1.99 ± 0.20	35	2.15 ± 0.24	40	2.22 (0.39)
**Outcome variables**										
Technical Skill										
Modified AFKT score	18	12.0 ± 2.0	52	9.0 ± 4.0	25	12.0 ± 3.0	37	8.0 ± 3.0	41	5.0 ± 3.0
Modified AFHT score	19	5.0 ± 1.0	52	3.0 ± 1.0	29	4.0 ± 2.0	27	3.0 ± 1.0	38	2.0 ± 1.0
Sports injury										
Injured (%)	29	74.4	12	54.5	15	53.6	6	24.0	11	32.4
Not injured (%)	10	25.6	10	45.5	13	46.4	19	76.0	23	67.6

Normally distributed variables reported as mean ± SD and skewed data presented as median (IQR). All muscular strength and endurance measures, knee extension angle, Y-balance composite reach score, and 6 m hop time represent dominant side only. Y-balance anterior reach and running vertical jump tests indicative of non-dominant limb. ABD = abduction; AFHT = Australian football handballing test; AFKT = Australian football kicking test; AFL = Australian Football League; ANT = anterior; EXT = extension; FLEX = flexion; IR = internal rotation; VIES = visual inverse efficiency score

### Relationships between physical fitness and technical skill

Total sample and competition level subgroup stepwise multiple linear regression results of significant explanatory variables for kicking and handballing scores are shown in Table 5. After accounting for years of AF playing experience, increased running vertical jump height, greater hip abduction strength, and faster 6 m hop time accounted for 53.9% (adjusted *R*^*2*^ = 0.522, *p* < 0.001) of the variance in kicking accuracy scores. Quicker Australian Football League (AFL) agility time and greater lean mass explained 23.4% (adjusted *R*^*2*^ = 0.221, *p* < 0.001) of the variation in handballing accuracy scores. All physical fitness variables excluded from the models were deemed non-significant, demonstrating insufficient evidence of a relationship with mAFKT and mAFHT scores.

**Table 5:** Stepwise multiple linear regression results for the significant relationships between physical fitness characteristics and modified Australian football kicking and handballing tests scores.

Explanatory variables	Sample size (*n*)	R^2^	Adjusted *R*^*2*^	Unstandardised Beta coefficient (95% CI)	Coefficient SE	Standardised Beta coefficient	*p*-value
*Modified Australian football kicking score*							
**Total sample**	134	0.539	0.522				<0.001
Running vertical jump (cm)				0.095 (0.036, 0.155)	0.030	0.285	0.002
Playing experience (years)				0.468 (0.223, 0.712)	0.123	0.259	<0.001
6 m hop (s)				-3.395 (-5.541, -1.250)	1.083	-0.258	0.002
Hip ABD strength (N)				0.030 (0.012, 0.048)	0.009	0.244	0.001
**Elite senior**	16	0.338	0.288				0.023
Running vertical jump (s)				0.127 (0.021, 0.234)	0.049	0.582	0.023
**Non-elite senior**	47	0.246	0.223				0.002
Y-balance composite reach (%)				25.260 (9.580, 40.940)	7.707	0.496	0.002
**High-level junior**	24	0.369	0.332				0.006
Knee EXT angle (deg)				0.189 (0.063, 0.316)	0.060	0.607	0.006
**Non-elite junior (10–13 years)**	39	0.578	0.549				<0.001
Knee EXT strength (N)				0.036 (0.022, 0.050)	0.007	0.636	<0.001
Body fat (%)				-0.217 (-0.352, -0.082)	0.066	-0.396	0.003
*Modified Australian football handballing score*							
**Total sample**	142	0.234	0.221				<0.001
AFL agility (s)				-0.634 (-1.002, -0.265)	0.186	-0.320	0.001
Lean mass (kg)				0.047 (0.011, 0.084)	0.018	0.241	0.012
**Non-elite senior**	47	0.376	0.337				<0.001
AFL agility (s)				-1.411 (-2.111, -0.710)	0.344	-0.607	<0.001
Y-balance anterior reach (cm)				-0.085 (-0.146, -0.024)	0.030	-0.419	0.008
**High-level junior**	28	0.225	0.179				0.040
Lean mass (kg)				-0.260 (-0.506, -0.013)	0.117	-0.474	0.040

There were no explanatory variables demonstrating a statistically significant relationship with kicking score for non-elite junior (14–17 years) competition level or with handballing score for elite seniors and non-elite junior (14–17 years) competition levels. Age was a significant confounder demonstrating a relationship with handballing score for non-elite juniors (10–13 years). All muscular strength measures, knee extension angle, and 6 m hop time represent dominant side only. Running vertical jump and Y-balance anterior reach indicative of non-dominant limb. ABD = abduction; AFL = Australian Football League; EXT = extension.

### Relationships between physical fitness and sports injury

**Fig 1:**
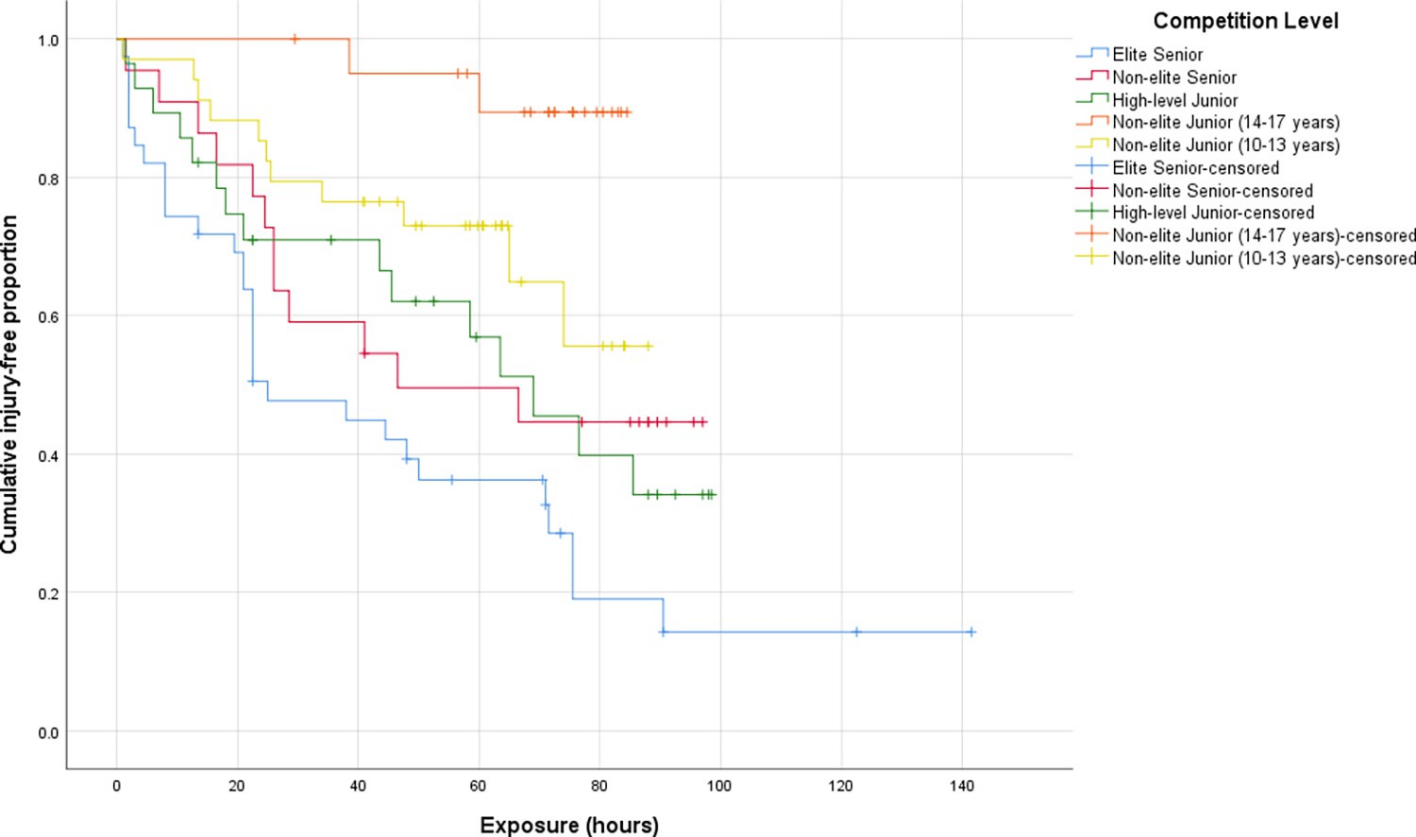
Kaplan-Meier survival curves for participating in training and matches free of time-loss injury in female Australian football players across five competition levels.

During the study period, 73 (49.3%) players sustained a time-loss AF injury in their respective competitive season. Most of these injuries occurred during matches (n = 54, 74.0%) compared to trainings (n = 19, 26.0%). The lower extremity was the most injured body region (n = 40; 54.8%), followed by the head and neck (n = 13; 17.8%), upper extremity (n = 12, 16.4%), and torso (n = 8, 11.0%). Time to injury was unable to be calculated for four, injured non-elite junior (14–17 years) players as date of injury was not provided; therefore, these players were excluded in the Kaplan-Meier survival curve analysis. Kaplan-Meier survival curves for time-loss, in-season AF injuries across five competition levels are shown in Fig 1. Elite senior players had a median time to injury of 25.0 (95% CI 10.7, 39.3) exposure hours, whereas non-elite senior and high-level junior players had a median time to injury of 46.5 (95% CI 0.0, 101.8) and 69.0 (95% CI 46.7, 91.4) exposure hours, respectively. The median survival for both non-elite junior competition levels were undefined. Table 6 provides the cut-off values for the categorical groupings of the potential risk factors utilised in the Cox regression analyses.

**Table 6:** Cut-off values for the categorical groupings of the potential risk factors utilised in the Cox regression analyses.

Potential risk factors	Category	Cut-off values				
		Elite senior	Non-elite senior	High-level junior	Non-elite junior (14–17 years)	Non-elite junior (10–13 years)
**Body composition**						
Body fat (%)	Above/below median	27.7	28.6	29.1	31.5	29.9
Lean mass (kg)	Above/below median	46.6	44.5	41.7	38.5	31.4
**Muscular strength and endurance**						
Shoulder IR strength (N)	Above/below median	156.0	140.0	140.0	102.3	84.5
Hip ABD strength (N)	Above/below median	147.0	111.2	133.4	94.6	70.4
Knee EXT strength (N)	Above/below median	333.6	282.5	302.5	305.0	211.0
Knee FLEX strength (N)	Above/below median	146.8	115.6	137.9	132.0	100.0
Side bridge (s)	Above/below median	102.0	69.5	70.5	59.0	52.0
**Flexibility**						
Knee EXT angle (deg)	Tight/Normal	20	20	20	20	20
**Balance**						
Y-balance ANT reach distance (cm)	Above/below median	60.0	61.0	60.3	62.3	61.0
Y-balance composite reach score (%)	Above/below median	103.3	101.6	98.2	104.3	99.3
**Reaction time**						
VIES (ms)	Above/below median	356.3	387.5	376.0	397.0	434.0
**Whole-body locomotor performance**						
AFL agility (s)	Above/below median	8.97	8.98	8.90	9.78	10.15
20 m sprint (s)	Above/below median	3.36	3.48	3.39	3.58	3.68
Running vertical jump (cm)	Above/below median	51.0	45.0	51.0	40.0	33.5
6 m hop (s)	Above/below median	1.94	2.07	1.96	2.13	2.22

All muscular strength and endurance measures, knee extension angle, Y-balance composite reach score, and 6 m hop time represent dominant side only. Y-balance anterior reach and running vertical jump tests indicative of non-dominant limb. ABD = abduction; AFL = Australian Football League; ANT = anterior; EXT = extension; FLEX = flexion; IR = internal rotation; VIES = visual inverse efficiency score.

Hazard ratios from the univariate Cox regressions for each physical fitness characteristic as a risk factor for time-loss AF injury are shown in Table 7. Univariate Cox regression analyses revealed four physical fitness variables had a significant relationship with time-loss injury. Players with below median hip abduction, knee extension, and knee flexion strength had a reduced risk of injury. In other words, those possessing weaker hip abduction, knee extension, and knee flexion strength had a 58%, 42%, and 44% reduced risk of time-loss injury, respectively, compared to players with greater lower extremity strength. Conversely, players with above median AFL agility time had an increased risk of sustaining a time-loss injury, indicating those who are less agile had almost twice the risk of early injury, compared to more agile players.

**Table 7:** Univariate Cox regression results examining the relationships between physical fitness characteristics and injury in female Australian football players.

Explanatory variables	Category	Univariate HR (95% CI)	*p*-value
**Body composition**			
Body fat (%)	Above median	1.03 (0.61, 1.74)	0.911
Lean mass (kg)	Below median	0.89 (0.53, 1.50)	0.656
**Muscular strength and endurance**			
Shoulder IR strength (N)	Below median	0.72 (0.44, 1.15)	0.170
Hip ABD strength (N)	Below median	0.42 (0.26, 0.70)	0.001*
Knee EXT strength (N)	Below median	0.58 (0.35, 0.97)	0.036*
Knee FLEX strength (N)	Below median	0.56 (0.34, 0.93)	0.023*
Side bridge (s)	Below median	0.81 (0.49, 1.35)	0.421
**Flexibility**			
Knee EXT angle (deg)	Tight	0.81 (0.47, 1.42)	0.464
**Balance**			
Y-balance ANT reach distance (cm)	Below median	0.74 (0.46, 1.19)	0.213
Y-balance composite reach score (%)	Below median	1.21 (0.75, 1.95)	0.446
**Reaction time**			
VIES (ms)	Above median	0.62 (0.38, 1.03)	0.064
**Whole-body locomotor performance**			
AFL agility (s)	Above median	1.91 (1.01, 3.60)	0.046*
20 m sprint (s)	Above median	0.99 (0.55, 1.78)	0.971
Running vertical jump (cm)	Below median	1.24 (0.70, 2.21)	0.462
6 m hop (s)	Above median	0.96 (0.51, 1.82)	0.907

* Indicates statistical significance (*p* < 0.05).

All muscular strength and endurance measures, knee extension angle, Y-balance composite reach score, and 6 m hop time represent dominant side only. Y-balance anterior reach and running vertical jump tests indicative of non-dominant limb. ABD = abduction; AFL = Australian Football League; ANT = anterior; EXT = extension; FLEX = flexion; IR = internal rotation; VIES = visual inverse efficiency score.

When considering hip abduction (adjusted HR 0.55, 95% CI 0.27, 1.13, *p* = 0.102), knee extension (adjusted HR 0.54, 95% CI 0.25, 1.16, *p* = 0.113), and knee flexion (adjusted HR 0.82, 95% CI 0.39, 1.74, *p* = 0.604) strength collectively with AFL agility time in a multivariate model, only AFL agility time remained significant (adjusted HR 2.41, 95% CI 1.23, 4.73, *p* = 0.010). However, when adjusted for age and years of AF experience, this no longer remained as a significant physical fitness risk factor of time-loss, in-season injuries in female AF players (adjusted HR 1.68, 95% CI 0.81, 3.50, *p* = 0.166).

## Discussion

The aims of this study were to explore the relationships between physical fitness and i) technical skill attributes and ii) time-loss injury in female AF players. After accounting for years of playing experience, increased running vertical jump height, greater hip abduction strength, and faster 6 m hop time accounted for 53.9% of the variance in kicking accuracy scores, whereas faster AFL agility time and greater lean mass were found to be significantly related to handballing accuracy score, explaining 23.4% of the variance. Competition level analyses revealed that greater knee extension strength and decreased body fat percentage were associated with kicking accuracy in non-elite juniors (10–13 years), with few significant relationships between physical fitness characteristics and kicking and handballing accuracy in the other competition levels. A somewhat surprising finding was that players with weaker hip abduction, knee extension, and knee flexion strength had a reduced risk of injury. Conversely, less agile players had an increased risk of sustaining a time-loss injury. However, after controlling for age and years of playing experience, no relationships were demonstrated between physical fitness characteristics and injury risk in female AF players, although this could partially be due to limited sample size and power.

### Relationships between physical fitness and technical skill

Collectively, the physical fitness measures demonstrating a relationship with kicking accuracy in this study represent strength, power, and coordination of the lower extremity, suggesting a stable support leg may allow for better distal mobility of the swing leg, enabling greater kicking accuracy [47]. Specifically, greater stabilisation through the support leg and ability to lift the whole-body upwards facilitates better control and capacity to generate faster foot speeds during the swing phase of the kicking leg [47–49]. Research in male AF has also demonstrated greater non-dominant versus dominant running vertical jump height, possibly reflecting the importance of dynamic balance and power in the non-dominant stance leg while kicking on the run during game play [50]. As such, the role and movement pattern of the non-dominant stance leg during the kicking action may be of importance in developing this technical skill, however future research is needed to better understand this relationship when considering various task constraints (e.g., goal kicking from a set shot vs. field kicking) specific to female competitions [49].

Competition level analyses revealed that greater knee extension strength and decreased body fat percentage were associated with kicking accuracy in non-elite juniors (10–13 years). These findings may suggest those junior players with greater quadriceps strength were better able to apply sufficient force to kick the football the distances (15 m, 20 m, and 25 m) required of the test used in this study. To meet the demands of greater distances, players are required to increase foot and ball speed, accordingly [51], whereas decreased foot speed has been demonstrated in more accurate kicks [49, 52]. Therefore, the findings in this study in which players with stronger quadriceps had better accuracy may reflect the speed/distance-accuracy trade-off, whereby stronger individuals with a greater maximal kicking speed/distance are more likely to be accurate at kick distances approaching maximal for the weaker players. This finding is similar to that of Hart and colleagues [26], which found sub-elite senior male AF players with greater lower limb strength had better kicking accuracy. Similarly, the sub-elite senior male accurate kickers also possessed significantly lower relative fat mass [26], a finding consistent with the non-elite juniors (10–13 years) in this study. There is a complex interaction of many body segments that is required for skilled kicking [47] and previous research has concluded that measures of adiposity, such as body fat percentage, were negatively correlated with motor coordination in children and adolescents [53]. Thus, decreased body fat percentage may enhance a developing kicker’s ability to coordinate their limbs by achieving better positioning on their stance leg and movement of the swing leg during the kicking action. Conversely, one study in elite senior female AF players (AFLW) reported no significant differences for body composition characteristics and kicking efficiency [54]. This may be explained by biological maturation influences on kinanthropometric variables and physical fitness attributes in young athletes, with early maturation often demonstrating higher kinanthropometric values and better physical fitness performance results [55]. While maturation status was not investigated in this study, it may also influence the relationship between body fat percentage and kicking skill seen in the youngest cohort of this study. These findings allude to the importance of selected physical fitness characteristics in junior skill development, but further research is needed to better understand this relationship across the different levels of female AF and considering maturational status.

Results showed that faster AFL agility times and greater lean mass were associated with better handballing accuracy in female AF players. These findings may be explained by opportunities the game affords, with players who are exposed to congested playing situations having more opportunities to perform more handballs. For example, players who are more agile and have a greater proportion of lean muscle mass are more likely to engage in repeated, congested, ball contesting scenarios. One study in elite men’s AF revealed 68% of handball executions occurred in the midfield, 44% occurred when the player was in-motion (run or jog), and 46% of passes were performed under moderate or high pressure from the opposition [56]. In these situations, the player may be more likely to experience physical contact by opposition players, meaning they require the ability to quickly change direction and accurately handball the football to a teammate before getting tackled. Such an environment may afford opportunities for more agile and muscular players to develop their handball skills in game play. Further research is required to indicate typical handballing distances conducted within female competitions across the developmental pathway and to validate handballing tests in this athlete population.

### Relationships between physical fitness and sports injury

This study is the first to investigate physical fitness characteristics as potential risk factors for sports injury in female AF players across key competition levels. Univariate analyses revealed players with weaker hip abduction, knee extension, and knee flexion strength were found to have a reduced risk ratio to time-loss injury compared to those with greater muscular strength. These findings were initially surprising, as factors such as increased strength have typically been reported to be associated with reduced risk of injury in male athletes, including those playing AF [57]. Given a higher frequency of contact-related injuries has been reported in female AF across key competition levels [31], the results reported in this study may reflect the infancy of female competitions; those players possessing decreased strength may be less likely to get involved in high-impact body contact situations during game play and therefore, less likely exposed to inciting events resulting in an injury. As positional role differences regarding technical game actions have been reported within the AFLW [58], this may also be a factor impacting involvement in congested and high-impact game play. Future research to include physical fitness measures across positional groups in female AF players may provide greater insights regarding these relationships.

Conversely, less agile players had almost twice the risk of sustaining a time-loss injury compared to more agile players. This finding is consistent with research indicating change-of-direction manoeuvres are associated with sports injury, such as ACL injury [59]. Notably, sidestep cutting movements were the most prevalent (n = 11, 52.4%) in a video analysis of 21 ACL injuries sustained during a match from the 2017–2020 AFLW seasons [39]. This may be explained by an angle-velocity trade-off, by which faster approaches (i.e., velocity) may compromise the execution of movement (i.e., angle of direction change), which is influenced by an athlete’s physical fitness [60]. Therefore, less agile athletes may not possess the physical capacity required to mediate this trade-off, resulting in greater associative knee joint loading [60]. Additionally, less agile players may be less effective at evading contact-related situations, thereby exposed to more inciting events (such as tackles or contact with another player during competitive play) that may result in injury. Further research is warranted to better understand the role of physical fitness and its interaction with biomechanical strategies performed and mechanisms of injury within this rapidly developing athlete population to support the development of injury prevention strategies.

It is of note that when the univariate results were considered collectively in a multivariate analysis, these physical fitness characteristics were no longer deemed significantly related to time to injury. This reflects similar findings concluded in a systematic review investigating the relationship between physical fitness and sports injury in female team ball sport players [61]. However, the findings from this study must be interpreted with caution. Specifically, the number of injury events is limited in relation to the number of parameters utilised in the multivariate model, indicating it is under powered. Additionally, these findings may suggest that utilising reductionist methods to examine a complex phenomenon, such as injury causation, may not be appropriate [37]. While this study provides a foundation to support future research, transitioning to larger studies that investigate holistic interactions between a web of determinants (such as physical fitness, perceptual capabilities, history of previous injury, and psychological factors) in relation with environmental factors to determine injury patterns or regularities is warranted [37].

### Strengths and limitations

This is the first study to explore relationships between physical fitness, technical skill, and sports injury in female AF players across competition levels. These results, therefore, provide some preliminary insight to support practitioners regarding talent development and injury prevention in this population. A strength of this study is the use of a comprehensive physical fitness profile as explanatory variables of key AF technical skills and as potential risk factors for injury. However, this study is not without limitations and results should be interpreted with some caution. Firstly, competition level analyses largely revealed minimal relationships between physical fitness characteristics and kicking and handballing accuracy. This may be due to small sample sizes utilised in this study and the amount of missing data. Secondly, while the original kicking and handballing tests were validated in elite junior male AF players [62], the psychometric properties of the modified versions used in this study have not been examined in female AF players. Minimal relationships between physical fitness characteristics and time to sports injuries were also found in this exploratory study. This may be explained by the limited number of injured cases reported in this study, which may restrict our ability to detect small to moderate associations (requiring approximately 200 injured subjects) [44]. Additionally, the limited number of injured cases restricted a detailed analysis of the relationship of physical fitness measures with specific injury types, body regions, or injuries due to specific mechanisms. As such, it is unclear whether certain physical fitness characteristics impact specific injury priorities in female AF. Lastly, due to limited sample size, it was only possible to categorise potential physical fitness risk factors into dichotomous variables based on groups split by the median in the absence of known thresholds. Therefore, such analyses could not explore potential curvilinear effects.

Given these limitations, this study provides initial insight into skill development and injury prevention for female AF and offers a reference to which future studies can expand upon these findings to better support this population. Specifically, future research should further validate commonly utilised physical fitness and technical skill tests in female AF, as well as incorporate technical skill tests more representative of female AF game play than the kicking and handball accuracy tests used in the present study. Larger studies incorporating complex system analyses are needed that are inclusive of holistic, transdisciplinary data collection and sharing [63]. As such, wider collaboration amongst researchers is recommended to address technical skill development and injury prevention on both micro (i.e., organism) and macro (i.e., sociocultural) scales [63]. The creation of Prep to Play PRO acknowledges a socioecological approach [32] and the findings from this study may provide some support for the inclusion of movement skills, specifically change of direction, within the program. The findings from this study may also indicate that physical fitness may play another role in the web of determinants, with other elements of the program, such as education and football-specific preparation, might have a more integral role in injury risk reduction in female AF players. Additionally, as current junior players advance to senior competition levels, physical fitness characteristics, technical skill attributes, and implications on sports injury are likely to change. The highly anticipated results from the undergoing stepped-wedge, cluster randomised control trial investigating the implementation of Prep to Play in U16, U18, and senior women’s competitions will likely provide further insights [33]. Regardless, further research is needed to support this rapidly evolving population to enhance talent development pathways and injury risk reduction programs.

### Conclusion

This study explored the relationships between physical fitness characteristics, kicking and handballing accuracy, and time-loss injury in female AF players. Significant relationships were found indicating players with higher running vertical jump height, greater hip abduction strength, and better lower extremity coordination demonstrated better kicking accuracy. More agile and muscular female AF players demonstrated better handballing accuracy. These results may assist sport practitioners regarding technical skill development in female AF players. Regarding injury, minimal relationships were found between physical fitness characteristics and time-loss injury when adjusted for age and years of playing experience. Future research is needed to expand upon these findings to better understand risk profile patterns in female AF players to support injury prevention programs.
